# PME-1-regulated neural cell death: new therapeutic opportunities?

**DOI:** 10.18632/aging.205303

**Published:** 2023-11-09

**Authors:** Liesbeth Guffens, Rita Derua, Veerle Janssens

**Affiliations:** 1 Laboratory of Protein Phosphorylation and Proteomics, Department of Cellular and Molecular Medicine, University of Leuven, KU Leuven, Belgium

**Keywords:** necroptosis, oxidative stress, neurodegeneration, PP2A, carboxymethylation

Alzheimer’s disease (AD), affecting 50 million individuals globally in 2018, stands as the primary cause of dementia. This neurodegenerative disorder is characterized by a range of molecular and cellular changes that contribute to neuronal cell death and the progressive decline in cognitive function and memory. In addition to pivotal molecular and cellular hallmarks defining AD, such as the buildup of Aβ plaques, the aggregation of tau proteins into neurofibrillary tangles, and neuroinflammation involving activation of glial cells, AD is characterized by increased oxidative stress [1]. Reactive oxygen species (ROS) levels have been shown to increase upon aging, affecting the entire body, with the central nervous system being particularly susceptible. While ROS are presumably not the triggering factor of AD, they are likely to contribute to the progression of the disease. For instance, excessive ROS production can exacerbate the accumulation and deposition of Aβ in AD. However, the precise mechanisms through which ROS participate in the pathogenesis of neurodegeneration remain elusive.

Here, we highlight a potential novel mechanism through which oxidative stress may drive the progression of AD, while we also explore how this mechanism may serve as a rationale for testing prospective AD therapies.

In a recent paper, we presented evidence that by inducing oxidative stress in two independent glioblastoma (GBM) cell lines – the pleomorphic/astrocytoid U251MG and the epithelial-like U87MG cell line – necroptosis was triggered via phosphorylation of MAP kinase-activated protein kinase 2 (MAPKAPK2) and receptor-interacting serine/threonine-protein kinase 1 (RIPK1). Notably, significantly increased necroptosis was observed when the GBM cells expressed higher levels of Protein Phosphatase 2A Methyl Esterase-1 (PME-1) [2], a protein responsible for the inactivation and demethylation of the widely expressed Ser/Thr protein phosphatase PP2A. PP2A phosphatases are holoenzymes consisting of three subunits: a catalytic PP2A-C subunit that is the direct substrate of PME-1, a structural PP2A-A subunit, and one of many regulatory B subunits that determine PP2A substrate specificity [3]. In the described context, PME-1 specifically inhibited the nuclear fraction of PP2A-B55α, a PP2A trimeric holoenzyme encompassing the B55α regulatory subunit, thereby inhibiting MAPKAPK2 dephosphorylation, and indirectly, resulting in increased activation of RIPK1 and increased cell death [2].

**Figure 1:**
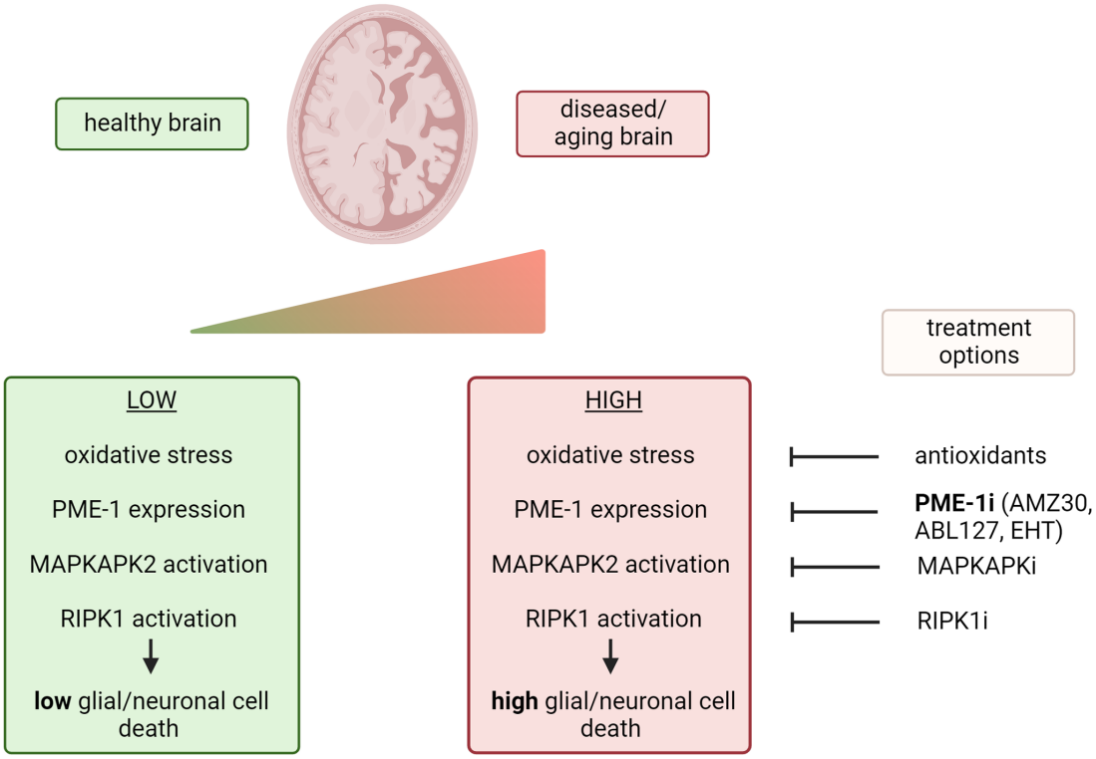
**Possible therapeutic options to prevent glial and/or neuronal cell death induced by oxidative stress.** i = pharmacologic inhibitor. Created with https://www.biorender.com/.

Considering that increased expression of PME-1 and increased demethylation of PP2A have been found in brain tissue of individuals with AD [4], and PP2A methylation has been shown to decrease in the aging brain [5], these findings could hold clinical significance for AD treatment or other age-related brain disorders. Although the ROS-induced necroptosis was demonstrated in non-neuronal brain tumor cells [2], we speculate that this mechanism may extend to non-tumoral glial cells and neuronal cells. Despite being less extensively studied, glial cells have been implicated in AD pathogenesis. Given the elevated ROS and PME-1 levels detected in AD brain tissue in contrast to healthy brain tissue, we hypothesize that these may contribute to the increased neuronal death observed in AD brain tissue, via increased PP2A-B55α inhibition and increased activation of the MAPKAPK2-RIPK1 pathway (Figure 1).

Consequently, the discovery of this signaling pathway may unveil several new avenues for targeted therapeutic intervention (Figure 1). In particular, inhibiting PME-1 itself emerges as a promising tool to slow down AD progression and its associated symptoms by mitigating oxidative stress-induced necroptosis. Two structurally unrelated classes of covalent inhibitors of PME-1 have already been developed for this purpose [6]: ABL127 and AMZ30. Although these inhibitors have not yet been formally assessed in an *in vivo* AD context, other natural products that inhibit PME-1 have already been successfully tested. For instance, the dietary supplementation of eicosanoyl-5-hydroxytryptamide (EHT), a minor component of coffee, decreased cognitive impairment and tau hyperphosphorylation, and decreased the cytoplasmic levels of β-amyloid protein in a rat model of AD [7]. EHT is known to bind to PP2A, thereby blocking binding of PME-1 [8]. While antioxidants [1], MAPKAPK2 inhibitors (Srivastava et al. 2021 – Expert Opin. Ther. Targets), and RIPK1 inhibitors (Li et al. 2022 – Pharmacol. Ther.) have been previously tested for AD treatment with mixed results, their effects might be more pronounced in patients with increased PME-1 levels – implying PME-1 expression as a potential predictive biomarker for these alternative treatments. However, the direct inhibition of PME-1 itself, which is more upstream in the cell death signaling pathway, may hold greater promise due to its potential additional downstream consequences on MAPKAPK2 and RIPK1 activity. In addition, since PME-1, via inhibition of PP2A-B55 (the main tau phosphatase), has also been described to increase tau and APP phosphorylation in transgenic mice [7], direct PME-1 inhibition could have the additional benefit of preventing neurofibrillary tangle and Aβ plaque formation during AD pathogenesis.
